# A biallelic variant of the RNA exosome gene, *EXOSC4*, associated with neurodevelopmental defects impairs RNA exosome function and translation

**DOI:** 10.1016/j.jbc.2024.107571

**Published:** 2024-07-14

**Authors:** Milo B. Fasken, Sara W. Leung, Lauryn A. Cureton, Maha Al-Awadi, Adila Al-Kindy, Ambro van Hoof, Sohail Khoshnevis, Homa Ghalei, Almundher Al-Maawali, Anita H. Corbett

**Affiliations:** 1 Department of Biology, Emory College of Arts and Sciences, Atlanta, Georgia USA; 2 Department of Biochemistry, Emory University School of Medicine, Atlanta, Georgia USA; 3 Graduate Program in Genetics and Molecular Biology, Emory University, Atlanta, Georgia, USA; 4 Sultan Qaboos Hospital, Ministry of Health, Salalah, Oman; 5 Department of Genetics, College of Medicine and Health Sciences, Sultan Qaboos University, Muscat, Oman; 6 Department of Microbiology and Molecular Genetics, The University of Texas Health Science Center at Houston, Houston, Texas, USA; 7 Sultan Qaboos University Hospital, Sultan Qaboos University, Muscat, Oman

**Keywords:** RNA exosome, EXOSC4, RNA processing/decay, translation, neurodevelopmental disorder

## Abstract

The RNA exosome is an evolutionarily conserved complex required for both precise RNA processing and decay. Pathogenic variants in *EXOSC* genes, which encode structural subunits of this complex, are linked to several autosomal recessive disorders. Here, we describe a missense allele of the *EXOSC4* gene that causes a collection of clinical features in two affected siblings. This missense variant (NM_019037.3: exon3:c.560T>C) changes a leucine residue within a conserved region of EXOSC4 to proline (p.Leu187Pro). The two affected individuals show prenatal growth restriction, failure to thrive, global developmental delay, intracerebral and basal ganglia calcifications, and kidney failure. Homozygosity for the damaging variant was identified by exome sequencing with Sanger sequencing to confirm segregation. To explore the functional consequences of this amino acid change, we modeled EXOSC4-L187P in the corresponding budding yeast protein, Rrp41 (Rrp41-L187P). Cells that express Rrp41-L187P as the sole copy of the essential Rrp41 protein show growth defects. Steady-state levels of both Rrp41-L187P and EXOSC4-L187P are decreased compared to controls, and EXOSC4-L187P shows decreased copurification with other RNA exosome subunits. RNA exosome target transcripts accumulate in *rrp41-L187P* cells, including the 7S precursor of 5.8S rRNA. Polysome profiles show a decrease in actively translating ribosomes in *rrp41-L187P* cells as compared to control cells with the incorporation of 7S pre-rRNA into polysomes. This work adds EXOSC4 to the structural subunits of the RNA exosome that have been linked to human disease and defines foundational molecular defects that could contribute to the adverse phenotypes caused by *EXOSC* pathogenic variants.

The RNA exosome is an evolutionarily conserved, ubiquitously expressed complex (1) composed of nine structural subunits and a catalytic 3′-5′ riboexonuclease/endonuclease (2, 3), which is required for essential processes, such as the production of mature rRNA and processing of noncoding RNAs (*e.g.*, snRNA, snoRNA) in the nucleus, degradation of aberrant RNAs in both the cytoplasm and the nucleus, and turnover of mRNAs in the cytoplasm (4, 5). The nine structural subunits of the complex consist of three cap subunits (EXOSC1-3) and six core subunits (EXOSC4-9) that form a barrel-like structure (6). These structural subunits interact with catalytic nucleases, DIS3 and/or EXOSC10 in the nucleus or DIS3L in the cytoplasm (7, 8, 9). RNA substrates are directed from the cap into the central channel of the complex to DIS3/DIS3L at the bottom of the complex and/or EXOSC10 at the cap for processing/decay (3). Both the individual subunits and the overall structure of this essential complex are evolutionarily conserved (10, 11, 12).

A number of pathogenic missense variants have been identified in genes that encode structural subunits of the RNA exosome complex (10). The initial finding was that mutations in the *EXOSC3* gene are linked to pontocerebellar hypoplasia type 1B (13). Subsequent studies have led to the identification of additional pathogenic variants in *EXOSC3* (14) as well as in *EXOSC1*, *EXOSC2*, *EXOSC5*, *EXOSC8*, and *EXOSC9* that have been linked to autosomal recessive disease (15, 16, 17, 18, 19, 20). The majority of the pathogenic variants identified in *EXOSC* genes cause single amino acid substitutions. The clinical presentation associated with these different variants are diverse (10); however, many of the variants impact the cerebellum at least to some extent, consistent with the link to pontocerebellar hypoplasia. How variants that impact one ubiquitously expressed, essential complex cause distinct pathology is not yet clear.

Studies seeking to understand how pathogenic missense variants in genes encoding structural subunits of the RNA exosome contribute to pathology have employed a variety of different approaches. A number of studies have analyzed cells derived from patients, primarily fibroblasts (17, 19, 20, 21). Other studies have employed genetic model systems, including budding yeast and *Drosophila* (16, 18, 22, 23, 24, 25, 26). Results from these studies suggest that the pathogenic variants typically do impair RNA exosome function, but the mechanisms by which the complex function is impaired remain largely elusive. Some studies demonstrate that these variants cause a decrease in the steady-state level of both the directly affected subunit and other associated subunits (20, 22), which could suggest an overall decrease in the level of the RNA exosome complex. However, the diversity of pathology observed in patients suggests that simple downregulation of the complex levels is not sufficient to explain disease mechanism. Indeed, evidence suggests that the specific amino acid substitution may alter the function or interactions of the affected subunits (18, 22). Identification of additional variants as well as analysis of how changes in the specific subunit levels impact function can begin to provide insight into how RNA exosome variants alter the function of the complex and confer diverse disease pathology.

Here, we report the identification of a novel, homozygous missense variant, p.L187P, in the RNA exosome gene *EXOSC4* that is linked to a neurodevelopmental disorder in two siblings. To explore the functional consequences of this amino acid substitution, we model this pathogenic variant in the corresponding budding yeast protein Rrp41. Cells that express Rrp41-L187P as the sole copy of the essential Rrp41 protein show growth defects. The steady-state level of the Rrp41-L187P protein is decreased relative to the level of WT Rrp41, suggesting the L187P amino acid change could alter protein stability. Consistent with these results, *rrp41-L187P* cells show significant accumulation of RNAs that are normally processed or degraded by the RNA exosome, including the 7S precursor of mature 5.8S rRNA as well as a reduction in the level of polysomes. We demonstrate that the decrease in RNA exosome function is not solely explained by the decrease in steady-state levels of the defective subunit in the budding yeast model. Extending the analysis into mammalian cells, steady-state levels of the mammalian EXOSC4-L187P protein are decreased relative to WT EXOSC4 in a neuronal cell line, suggesting that mechanisms defined in budding yeast may extend to the human protein. Finally, EXOSC4-L187P shows decreased copurification with other RNA exosome subunits, suggesting this EXOSC4 variant has decreased interaction with other subunits of the complex. This study adds EXOSC4 to the RNA exosome subunits that have now been linked to neurological deficits in humans and defines molecular consequences from this disease-causing *EXOSC* variant.

## Results

### Clinical report

**Figure 1:**
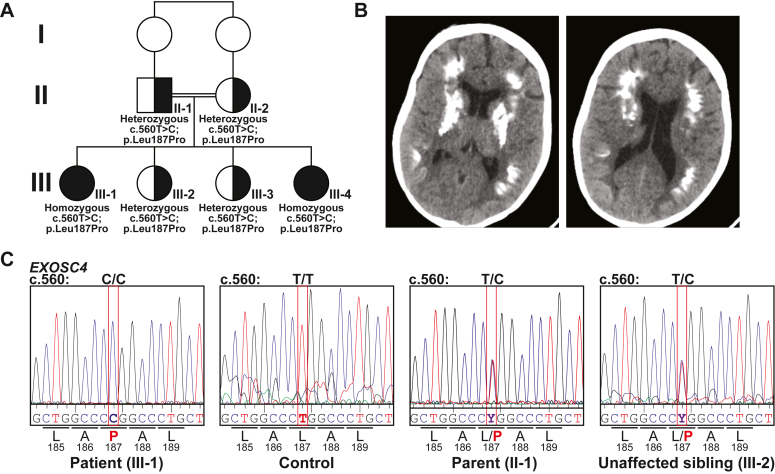
**Identification of an *EXOSC4* variant.***A*, pedigree of the Omani family with two affected offspring. Shaded symbols indicate affected individuals. The genotype is shown for those individuals who were analyzed. All genotypic carrier individuals are phenotypically healthy. *B*, axial CT brain scan for the affected individual III-1 at the age of 16 years shows bilateral diffuse calcification of the basal ganglia, including the caudate nucleus along with subcortical calcification of both parietal lobes at the centrum semiovale level. *C*, chromatograms for the Sanger sequencing showing the sequencing for patient (III-1), control, parent (II-1), and unaffected sibling (III-2). The sequencing identified the *EXOSC4* variant (NM_019037.3:c.560T>C (p.Leu187Pro)). The nucleotide sequence variant is indicated on *top* with the change in amino acid sequence indicated on the *bottom*.

Family 10DF16100 is an Omani family with two affected siblings born to consanguineous parents (Fig. 1). The index patient (III-1; Fig. 1*A*) is a female; she was the first child born to a 21-year-old primigravida mother, following a pregnancy complicated with a low volume of amniotic fluid and intra-uterine growth restriction. She was delivered *via* a planned Caesarean section at 36 weeks of gestation, with a birth weight of 2.06 kg (10th percentile), head circumference of 31.8 cm (10th percentile), and height of 41 cm (third percentile; −2.5 SD). She required no active resuscitation and had normal APAGR scores. She was admitted to the neonatology ward for 2 weeks to establish feeding and weight monitoring. The patient was first evaluated at our center at the age of 3 years where she presented with developmental delay and esotropia. Her developmental milestones assessment showed a global delay in all domains. She had head control at 8 months, sat at the age of 12 months, was able to stand with support at the age of 24 months, and was able to walk with support at the age of 3 years. She was able to reach, grasp, and transfer objects at the age of 3 years. She started babbling at the age of 1 year, but by three, she had not developed any words. At the age of three, she displayed significant stranger anxiety.

Her growth parameters at the age of three showed a weight of 8.95 kg (−3.7 SD), a head circumference of 46 cm (−1.6 SD), and a height of 80.5 cm (-3.5 SD). She showed progressive postnatal growth retardation. Her physical examination revealed frontal bossing, deep-seated eyes, retrognathia, and loss of facial subcutaneous fat as well as sparse hair. Her neurological examination showed axial hypotonia, all limbs spasticity with brisk deep tendon reflexes but absent clonus. Her systemic examinations, including the abdomen, heart, and skin, were unremarkable. Ophthalmology examination showed large angle esotropia with both eyes in the adduction position and no significant refractive error.

At the age of 10 years, she started to develop kidney failure; investigations did not reveal a specific cause. She later developed end stage kidney failure and was on regular dialysis. At the age of 12 years, her development milestones showed ability to walk with support only, although she could stand alone. She was able to hold pencils and scribble, but she could not draw or copy a circle. She was able to say two-word sentences and understand simple commands. She could count up to three and identify body parts and a few colors. She was not yet toilet trained. Her overall developmental stage was at ∼12 to 24 months. Her routine examinations revealed iron deficiency anemia, otherwise normal liver functions, bone profile, serum calcium and magnesium, thyroid function tests, karyotype, metabolic screen, urine organic acid, and other routine tests. Echocardiography, electrocardiogram, X-ray skeletal survey, nerve conduction studies, and ultrasound abdomen and pelvis and hearing test were all normal. The MRI brain at the age of 3 years showed mild generalized brain atrophy with no other abnormalities (data not shown). No other brain malformations were identified, and the ventricular system and posterior structures fossa appeared normal. A CT brain imaging at the age of 16 years showed bilateral diffuse calcification of the basal ganglia, including the caudate nucleus along with subcortical calcification of both parietal lobes at the centrum semiovale level (Fig. 1*B*). At the age of 19 years, she presented with history suggestive of acute brain hematoma. The CT scan brain confirmed a large and extensive acute bleed in the left basal ganglia with extension into left lateral, third, and fourth ventricles. Despite craniotomy and decompression, and intensive care, she passed away.

While this patient was under follow-up, a similarly affected sister (III-4; Fig. 1*A*) was delivered to this consanguineous couple at a different hospital. She presented with a similar phenotype. Her pregnancy and delivery history were unremarkable, and she was born at full term with normal birth parameters. At the first assessment at the age of 2 years, her growth parameters showed a weight of 9.3 kg (−2.5 SD), a head circumference of 47 cm (−0.33 SD), and a height of 80.5 cm (-1.5 SD). Her physical examination revealed deep-seated eyes and retrognathia with pointed chin. Her neurological examination showed axial hypotonia, all limbs spasticity with brisk deep tendon reflexes, but absent clonus. Her systemic examinations, including the abdomen, heart, and skin, were unremarkable. Ophthalmology examination showed bilateral esotropia and myopic astigmatism. At the age of 10 years, her growth metrics were a weight of 15.4 kg (−3.7 SD), a head circumference of 49 cm (−2.5 SD), and a height of 107 cm (−4.8 SD). At a developmental assessment at the age of 10 years, she was unable to walk without assistance, with an unsteady gait, and she had frequent falls. She could go up and down the stairs with support only. She could hold a pencil and scribble and copy a circle. She expressed her needs in 2–3-word sentences, but with limited vocabulary. She responded to her name and could count from 1 to 3 only and identify a few colors. However, the CT scan of the brain at the age of 13 months revealed a few tiny foci of hyperattenuation in both parietal lobes and in basal ganglia bilaterally (data not shown).

### Whole exome analysis

A targeted genetic investigation of the index patient (III-1) that included a next generation sequencing panel analyzing genes linked to DNA repair revealed no changes in any of the targeted genes (*DDB1, DDB2, ERCC1, ERCC2, ERCC3, ERCC4, ERCC5, ERCC6, ERCC8, GTF2H5, LIG4, MPLKIP, NHEJ1, POLH, UVSSA, XPA, XPC, XRCC4*). Thus, exome sequencing was performed to identify the genetic basis for this condition. The exome sequencing analysis of the two affected subjects along with the unaffected father revealed homozygosity for a variant in the *EXOSC4* gene in the affected subjects, but not in the father, that was confirmed by Sanger sequencing and cosegregates with the phenotype (Fig. 1*C*). The candidate variant (NM_019037.3:c.560T>C (p.[Leu187Pro])) in *EXOSC4* was the only variant shared between the two affected subjects that met the filtration criteria. Two siblings that do not share the clinical features of the affected siblings are not homozygous for this variant (Fig. 1*A*). Additionally, other genes known to cause neurodevelopmental delay, brain calcifications, or failure to thrive were analyzed and no other pathogenic variants were identified. The identified *EXOSC4* variant is absent from the publicly available variant databases (1000 Genomes, Exome Variant Server, and gnomAD) and the in-house control database. This *EXOSC4* (p.Leu187Pro) variant is predicted to be damaging/pathogenic by multiple *in silico* prediction tools, including BayesDel, MetaRNN, BayesDel noAF, REVEL GERP, LRT, MutationAssessor, MutationTaster, SIFT, and PROVEAN. This site (NM_019037.3:c.560T) is highly conserved among 100 vertebrate genomes (including humans).

### Functional consequences of the L187P amino acid change in EXOSC4

**Figure 2:**
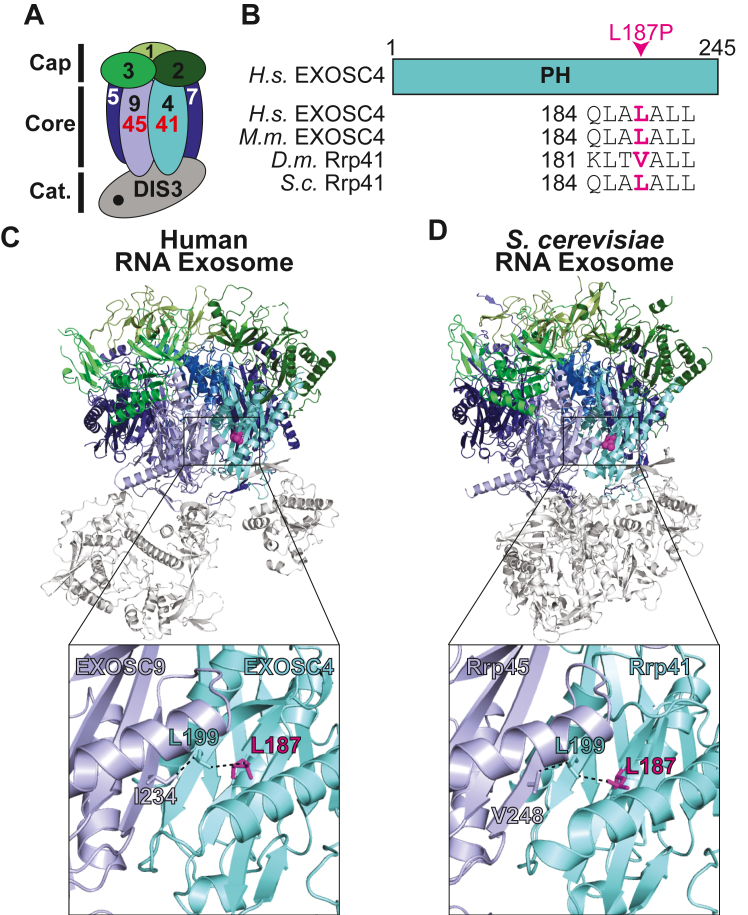
**Conservation and location of the Leu187 residue in the EXOSC4/Rrp41 subunit of the RNA exosome that is altered in disease.***A*, a cartoon model of the 10-subunit RNA exosome complex is shown. The subunits of the complex are indicated by their EXOSC number (1–3 in the cap, 4–9 in the barrel-like core ring), with the catalytic DIS3 subunit at the base. EXOSC4 (Rrp41 in budding yeast), which is a component of the barrel-shaped core ring structure, is highlighted in *teal* and the proximal subunit EXOSC9 (Rrp45 in budding yeast) is highlighted in *slate blue*. *B*, schematic of domain organization of EXOSC4. EXOSC4 is composed of a single RNase PH-like domain. Alignment of EXOSC4/Rrp41 amino acid sequences from *Homo sapiens* (*H.s.*), *Mus musculus* (*M.m.*), *Drosophila melanogaster* (*D.m.*), and *Saccharomyces cerevisiae* (*S.c.*) below the schematic show the conservation of the Leu187 residue (*magenta*). *C*, structural model of the human RNA exosome complex (PDB: 6D6R) (27) and (*D*) budding yeast RNA exosome complex (PDB: 6FSZ) (30). The zoomed in boxes in (*C* and *D*) depict the RNA exosome subunits EXOSC4/Rrp41 (*teal*) and EXOSC9/Rrp45 (*slate blue*) that share an interface and show the location of the Leu187 residue (*magenta*) in EXOSC4/Rrp41. The EXOSC4/Rrp41 residue Leu199 (*teal*) and the EXOSC9 residue Ile234/Rrp45 residue Val248 (*slate blue*) in close contact with the EXOSC4/Rrp41 residue Leu187 are shown.

As shown in Figure 2, the EXOSC4 Leu187 residue altered in the affected siblings is located in a highly conserved region of the EXOSC4 protein, which encodes a core subunit of the hexameric ring of the RNA exosome (Fig. 2, *A* and *B*). Within the three-dimensional structure of the RNA exosome complex, Leu187 lies in a region of EXOSC4 that is in close proximity to the interface with EXOSC9 (Fig. 2*C*). Specifically, Leu187 of EXOSC4, which is located in an α-helix, interacts with Leu199 of EXOSC4 within a neighboring β-strand that is in contact with Ile234 of EXOSC9. Similar types of contacts are formed in the yeast protein (Fig. 2*D*), Rrp41, where Leu187 interacts with Rrp41-L199, which then interacts with Val248 of Rrp45, the yeast ortholog of EXOSC9. Modeling of the L187P variant in a human RNA exosome structure (PDB ID: 6D6R) (27) using AlphaFold (28, 29) does not predict major structural changes for EXOSC4-L187P as compared to WT EXOSC4, but this analysis does predict disruption of the specific previously mentioned interactions that could result in the destabilization of EXOSC4 and affect interaction with EXOSC9.

**Figure 3:**
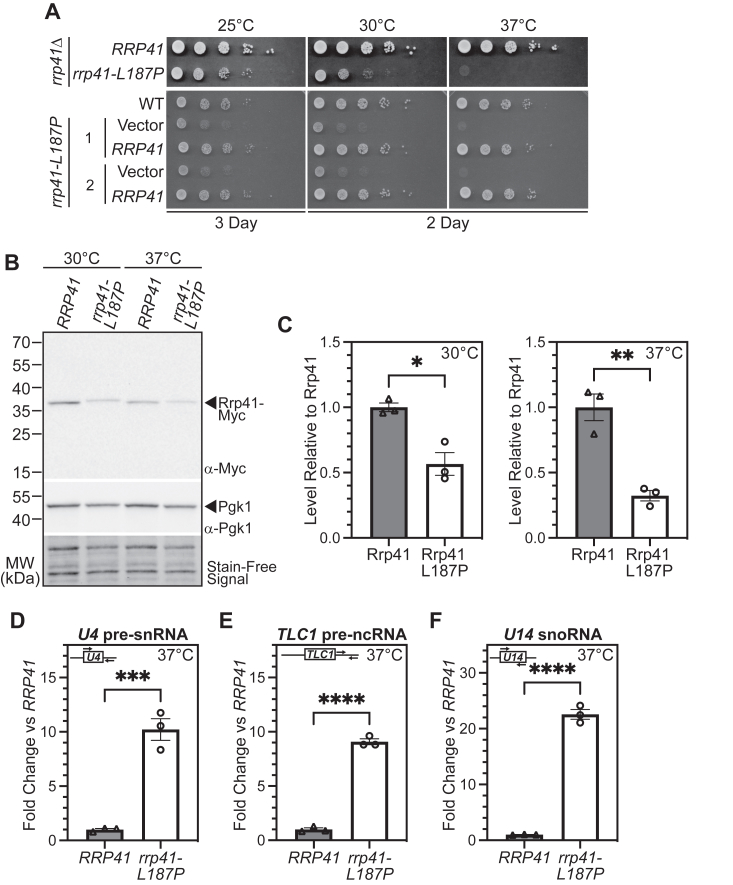
**The budding yeast *rrp41-L187P* mutant shows impaired growth, reduced Rrp41 protein level, and elevated levels of RNA exosome target transcripts.***A*, *rrp41-L187P* mutant cells show impaired growth at all temperatures (25, 30, 37 °C) tested. In the *upper panel*, the *rrp41Δ* cells containing *RRP41* or *rrp41-L187P* plasmid were serially diluted, spotted, and grown at the indicated temperatures for 2 to 3 days. In the *lower panel*, two *rrp41-L187P* CRISPR mutant strains (1 & 2) containing Vector or *RRP41* plasmid and a WT control were serially diluted, spotted, and grown at the indicated temperatures for 2 to 3 days. *B*, the steady-state level of the Rrp41-L187P protein variant is significantly decreased at 30 °C and 37 °C. Lysates of *rrp41Δ* cells solely expressing Myc-tagged, WT Rrp41, or Rrp41-L187P grown at 30 °C or 37 °C were analyzed by immunoblotting with an anti-Myc antibody to detect Rrp41-Myc and an anti-Pgk1 antibody to detect 3-phosphoglycerate kinase (Pgk1) as a loading control. The stain-free signal on the immunoblot is also shown as a loading control. *C*, quantitation of the relative level of Rrp41-L187P protein compared to Rrp41 detected in the lysates of *rrp41Δ* cells solely expressing Myc-tagged Rrp41 or Rrp41-L187P grown at 30 °C or 37 °C from three independent experiments – one shown in *B*. Graph shows the relative level of Rrp41-L187P-Myc protein compared to WT Rrp41-Myc, which is set to 1.0 (n = 3). Error bars represent SEM. Statistical significance is calculated by a Student’s *t* test (∗*p*-value ≤ 0.05; ∗∗ *p*-value ≤ 0.01). *D–F*, *rrp41-L187P* cells show elevated, steady-state levels of (*D*) *U4* pre-snRNA, (*E*) *TLC1* telomerase component pre-ncRNA, and (*F*) *U14* snoRNA relative to *RRP41* cells at 37 °C. Total RNA was isolated from *rrp41Δ* cells solely expressing *RRP41* or *rrp41-L187P* grown at 37 °C, and transcript levels were measured by RT-qPCR using gene-specific primers (illustrated above each graph), normalized relative to *RRP41*. Error bars represent SEM from three biological replicates. Statistical significance of the RNA levels in *rrp41-L187P* cells relative to *RRP41* cells was calculated by Student’s *t* test (∗∗∗*p*-value ≤ 0.001; ∗∗∗∗*p*-value ≤ 0.0001).

To explore the functional consequences of the L187P amino acid substitution in the EXOSC4 subunit of the RNA exosome, we modeled this pathogenic variant in the budding yeast ortholog of EXOSC4, Rrp41. As illustrated in Figure 2*B*, Leu187 is conserved between human EXOSC4 and budding yeast Rrp41. Furthermore, the conserved structure of the RNA exosome complex (30) means that Rrp41 Leu187 lies in close proximity to the interface with the core subunit that corresponds to EXOSC9, Rrp45 (Fig. 2*D*). The *rrp41-L187P* budding yeast cells were produced using either a plasmid shuffle or CRISPR genome-editing approach to express this Rrp41 variant as the sole copy of the essential Rrp41 protein. As an initial test of the function of Rrp41-L187P, we examined cell growth of these *rrp41-L187P* cells. Results of this analysis reveal that *rrp41-L187P* cells show growth defects at all temperatures examined with virtually no growth at 37 °C, severely impaired growth at 30 °C, and a mild growth defect even at 25 °C, as compared to control *RRP41* cells (Fig. 3*A*). For the cells generated by CRISPR genome editing (*rrp41-L187P* in Fig. 3*A*, lower panel), we confirmed that the growth defect is rescued when cells are transformed with WT *RRP41*, but not with the control Vector. To examine whether the L187P amino acid substitution alters the Rrp41 protein level, both WT Rrp41 and Rrp41-L187P were Myc-tagged and analyzed by immunoblotting. As shown in Figure 3*B* and quantitated in Figure 3*C*, Rrp41-L187P levels are decreased to 56% of control Rrp41 at 30 °C and 32% of control Rrp41 at 37 °C. We note a slight but reproducible shift in the migration of Rrp41-L187P compared to WT Rrp41. To assess the function of the RNA exosome in the *rrp41-L187P* cells, we examined the steady-state levels of documented small, noncoding transcript targets of the RNA exosome—*U4* pre-snRNA, *TLC1* pre-ncRNA, and *U14* snoRNA (31, 32, 33). For these three different RNA targets analyzed, there is significant accumulation (9–20-fold increase) of the RNA analyzed in *rrp41-L187P* cells as compared to *RRP41* cells (Fig. 3, *D*–*F*), suggesting that RNA exosome function is impaired in these cells.

**Figure 4:**
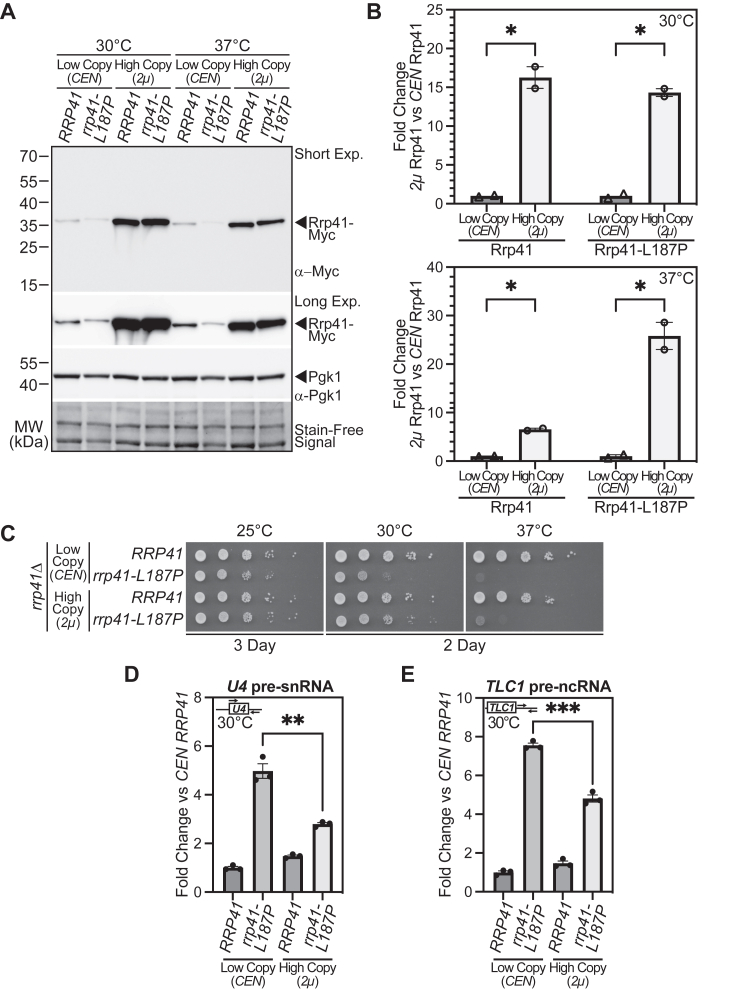
**Increasing the level of the Rrp41-L187P protein improves RNA exosome function but not to the same level as WT Rrp41**. Myc-tagged Rrp41 or Rrp41-L187P (*A* and *B*) or untagged Rrp41 or Rrp41-L187P (*C–E*) was expressed from either a low copy (*CEN*) or high copy (*2μ*) plasmid in *rrp41Δ* cells as the sole source of Rrp41 in the cells. *A*, as assessed by immunoblotting with an anti-Myc antibody, steady-state levels of both Rrp41-Myc and Rrp41-L187P-Myc are significantly increased from the high copy (*2μ*) plasmid compared to the low copy (*CEN*) plasmid. Samples were probed with anti-Pgk1 antibody to detect Pgk1 as a loading control. The stain-free signal on the immunoblot is also shown as a loading control. *B*, to quantitate the fold-change in levels of Rrp41 and Rrp41-L187P from high copy *versus* low copy plasmids, the level of high copy (*2μ*) Rrp41 or Rrp41-L187P was normalized to low copy (*CEN*) Rrp41 or Rrp41-L187P, respectively. Rrp41 protein expressed from the low copy (*CEN*) plasmid was therefore set to 1.0. As shown in the bar graphs, for results at 30 °C (*top*) and 37 °C (bottom), the levels of Rrp41 are increased 16.3-fold while levels of Rrp41-L187P are increased 14.3-fold for the high copy (*2μ*) plasmid *versus* the low copy (*CEN*) plasmid at 30 °C. Levels of Rrp41 are increased 6.5-fold while levels of Rrp41-L187P are increased 25.8-fold from the high copy (*2μ*) plasmid *versus* the low copy (*CEN*) plasmid at 37 °C. Error bars represent SEM. Statistical significance is calculated by a Student’s *t* test (∗*p*-value ≤ 0.05). *C*, the *rrp41Δ* cells expressing Rrp41-L187P from the high copy (*2μ*) plasmid show improved growth compared to cells expressing Rrp41-L187P from the low copy (*CEN*) plasmid at 25 °C and 30 °C. The *rrp41Δ* cells containing *RRP41* or *rrp41-L187P* low copy or high copy plasmid were serially diluted, spotted, and grown at the indicated temperatures for 2 to 3 days. *D and E*, the steady-state levels of RNA exosome target transcripts (*D*) *U4* pre-snRNA and (*E*) *TLC1* telomerase component pre-ncRNA exhibit a statistically significant decrease in *rrp41Δ* cells expressing Rrp41-L187P from the high copy (*2μ*) plasmid compared to cells expressing Rrp41-L187P from the low copy (*CEN*) plasmid. Total RNA was isolated from *rrp41Δ* cells expressing either low copy (*CEN*) or high copy (*2μ*) *RRP41* or *rrp41-L187P* plasmid grown at 30 °C, and transcript levels were measured by RT-qPCR using gene-specific primers (illustrated above each graph), normalized relative to the low copy (*CEN*) *RRP41* sample, which was set to 1.0. Error bars represent SEM from three biological replicates. Statistical significance was calculated by a Student’s *t* test (∗∗*p*-value ≤ 0.01; ∗∗∗*p*-value ≤ 0.001).

Given the decrease in the steady-state level of Rrp41 protein in the *rrp41-L187P* cells as well as previous work that demonstrated decreases in RNA exosome subunits in other exosomopathies (20), we designed an experiment to increase the level of the Rrp41-L187P variant and assess whether this increase in protein level could restore RNA exosome function. For this experiment, we expressed the Rrp41-L187P protein from a high copy (*2μ*) plasmid (34). As shown in Figure 4*A*, expression of both Rrp41 and Rrp41-L187P from this high copy plasmid leads to a substantial increase in the amount of protein produced relative to the low copy (*CEN*) plasmid. Quantitation of the protein levels shows that high copy (*2μ*) Rrp41 and Rrp41-L187P levels are increased 16.3-fold and 14.3-fold at 30 °C, respectively, and 6.5-fold and 25.8-fold at 37 °C, respectively, compared to low copy (*CEN*) Rrp41 levels (Fig. 4*B*). We then assessed the growth of cells that solely express Rrp41 or Rrp41-L187P at either low levels from the low copy (*CEN*) plasmid or high levels from the high copy (*2μ*) plasmid. At both 25 °C and 30 °C, growth of cells expressing high levels of Rrp41-L187P is improved compared to cells expressing low levels of Rrp41-L187P (Fig. 4*C*). Growth curve analysis of these cells at 30 °C confirms that cells expressing high (*2μ*) levels of Rrp41-L187P grow better than cells expressing low (*CEN*) levels of Rrp41-L187P, but these cells do not grow at the same rate as cells expressing WT Rrp41 (Fig. S1).

To explore the function of the RNA exosome under these conditions, we examined the steady-state levels of documented RNA exosome transcript targets (31, 32, 33). For each of these targets, there is a statistically significant decrease in the amount of RNA that accumulates in cells that express Rrp41-L187P from a high copy (*2μ*) plasmid as compared to the low copy (*CEN*) plasmid (Fig. 4, *D* and *E*). However, as for the growth phenotype, RNA exosome function is not fully restored. Taken together, these results suggest that a decrease in the steady-state level of RNA exosome subunit likely contributes to the loss of RNA exosome function in exosomopathies, but this decrease does not fully explain the loss of function, providing evidence that the specific amino acid substitution contributes to dysfunction and hence pathology.

### rrp41-L187P cells show defects in rRNA processing and polysome profiles

**Figure 5:**
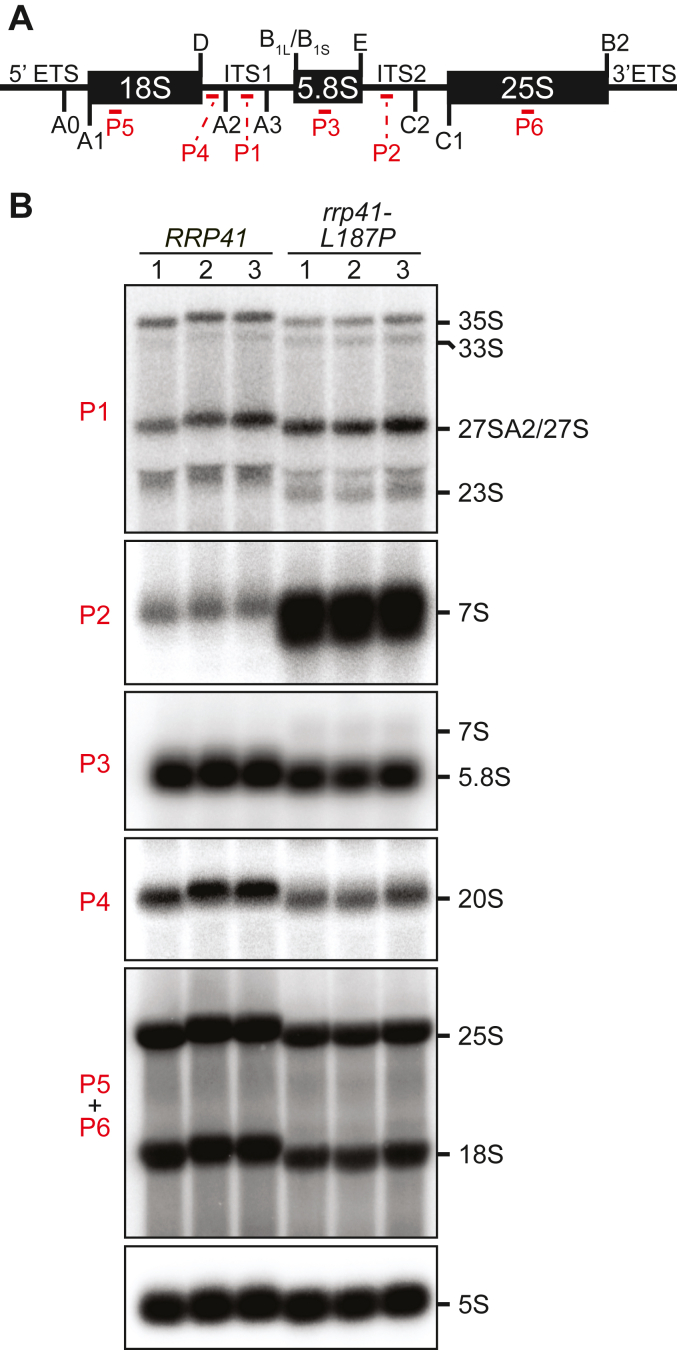
**The *rrp41-L187P* cells exhibit defects in rRNA processing**. *A*, schematic of yeast 35S rRNA indicating the locations of rRNA cleavage sites and northern blot probes used (P1-P6; *red*). The 35S rRNA transcript contains 18S, 5.8S, and 25S rRNA separated by internal transcribed spacer 1 and 2 (ITS1, ITS2) and flanked by 5′ and 3′ external transcribed spacer (5′ETS, 3′ETS). *B*, northern blotting of three independent biological replicates of *rrp41-L187P* CRISPR mutant and WT (*RRP41*) cells reveals rRNA processing defects in *rrp41-L187P* mutant cells, including the accumulation of 7S pre-rRNA. Equal amounts of total RNA from control WT *RRP41* cells or *rrp41-L187P* cells grown at 30 °C was used for northern blotting. The 5S probe was used a loading control.

As the RNA exosome is essential for the processing of ribosomal RNA (5, 35), we tested the extent to which rRNA processing is impacted in the CRISPR-engineered *rrp41-L187P* cells. For this analysis, we performed northern blotting and used a series of probes (P1-P6) to analyze the rRNA processing pathway (Fig. 5*A*). As shown for biological triplicates in Figure 5*B*, the *rrp41-L187P* cells show early rRNA processing defects resulting in accumulation of the 33S, 27S, and 23S pre-rRNA transcripts, with a concomitant decrease in the level of 20S pre-rRNA and accumulation of 7S pre-rRNA, the 5.8S precursor that is directly processed by the RNA exosome (36, 37). Northern blot analysis using *rrp41-L187P* cells generated by plasmid shuffle confirm these rRNA-processing defects (Fig. S2). Together, these data reveal a statistically significant increase in 7S rRNA and a decrease in 18S, 25S, and 5.8S rRNA levels (Fig. S2*C*).

**Figure 6:**
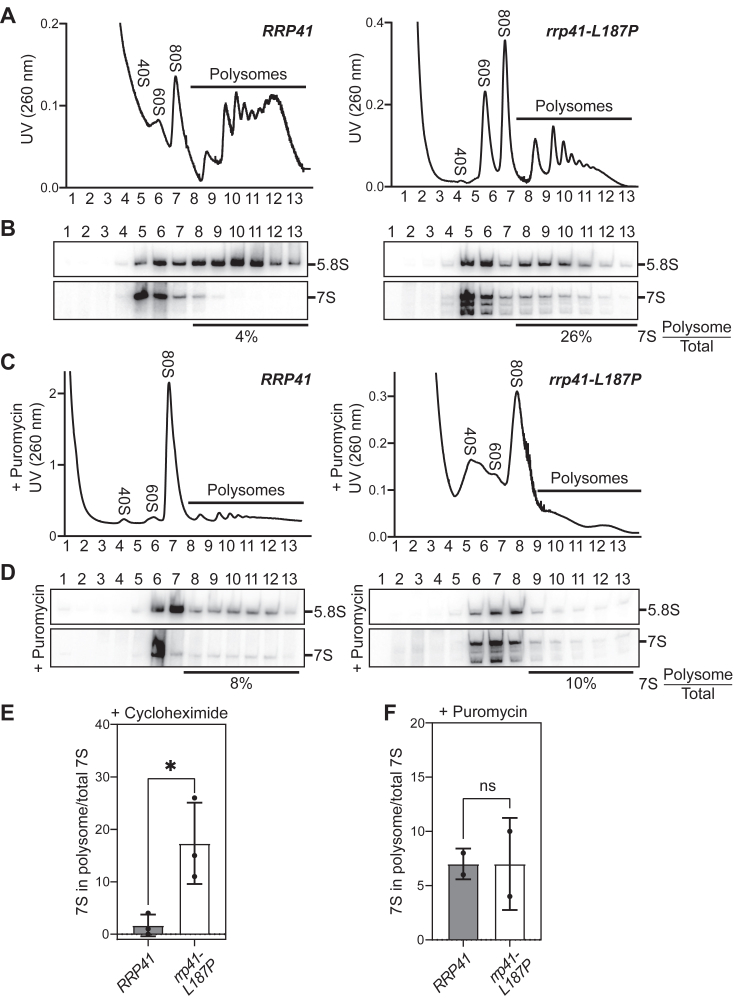
**The *rrp41-L187P* cells show altered polysome profiles**. *A*, polysome profiling reveals that *rrp41-L187P* CRISPR mutant cells show a loss of polysomes at 30 °C as compared to WT *RRP41* cells. Clarified cell extracts of *rrp41-L187P* CRISPR mutant cells and WT *RRP41* cells were resolved on a 10 to 50% sucrose gradient and scanned at 260 nm. The fraction numbers are indicated and peaks corresponding to the small (40S) and large (60S) ribosome subunits, as well as the monosomes (80S) and polysomes are marked. *B*, northern blotting on fractions from sucrose gradients treated with cycloheximide reveal the distribution of 5.8S rRNA and 7S pre-rRNA. The fraction of 7S rRNA present in polysomes was quantitated as described in Experimental procedures. *C*, to confirm that rRNA species detected by northern blotting in polysomes are not aggregates or other large complexes, samples were treated with puromycin prior to loading on the sucrose gradient and (*D*) fractions from these gradients were analyzed by northern blotting as described for (*B*). The fraction of 7S pre-rRNA migrating in polysomes *versus* total 7S pre-rRNA was quantified using data from three biological replicates for samples treated with cycloheximide (*E*) and two independent biological replicates for lysates treated with puromycin (*F*). Error bars represent SEM from biological replicates. Statistical significance was calculated by Student’s *t* test (∗*p*-value <0.05).

Given the defects in rRNA processing detected in *rrp41-L187P* cells, we performed polysome profiling to assess how translation is impacted in these cells (Fig. 6). Analyses of the polysome profiles reveal a marked reduction of polysomes in *rrp41-L187P* cells (Fig. 6*A*). Northern blot analysis of the polysome fractions reveals 7S rRNA-containing particles in polysome fractions of the *rrp41-L187P* mutant cells (Fig. 6*B*). To assess whether these 7S pre-rRNA containing complexes migrating in polysome fractions are aggregated complexes or premature subunits that escape into the translating ribosome pool, we omitted cycloheximide and added puromycin to lysates prior to analysis on gradients. Omission of cycloheximide from polysome analysis allows run off of the translating ribosomes, and puromycin dissociates translating ribosomes (38). Under these conditions, polysomes collapse into monosomes (Fig. 6*C*) and we observe that the bulk of 7S pre-rRNA and 5.8S rRNA shifts to the 80S and 60S fractions (Fig. 6*D*), demonstrating that a portion of 7S pre-rRNA found in the polysome fractions of the *rrp41-L187P* mutant cells is assembled into active ribosomes. We quantitated the fraction of 7S rRNA present in polysomes in three independent polysome-profiling experiments (Figs. 6*B* and S3) and found this value ranged from 11% to 26% for *rrp41-L187P* cells, as compared to 0 to 4% for WT *RRP41* cells, demonstrating a significant accumulation of 7S rRNA in polysomes in *rrp41-L187P* cells (Fig. 6*E*). In contrast, quantification of 7S pre-rRNA from gradients treated with puromycin reveals no significant change between WT *RRP41* and *rrp41-L187P* cells (Fig. 6*F*), as would be expected if the majority of 7S pre-rRNA in polysomes was associated with translating ribosomes that were dissociated in the presence of puromycin.

### Mammalian EXOSC4-L187P exhibits reduced steady-state levels and shows reduced interaction with other RNA exosome subunits

**Figure 7:**
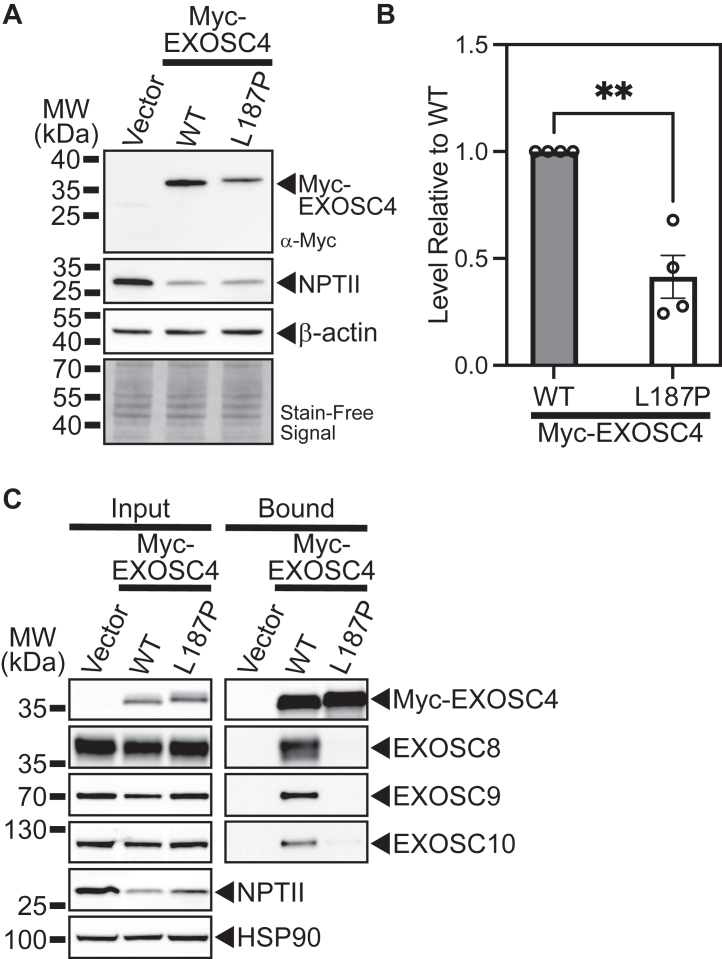
**The pathogenic amino acid substitution in EXOSC4decreases the steady-state level of the protein andcan alter interactions with other RNA exosome subunits.***A*, the murine EXOSC4-L187P variant, corresponding to the human EXOSC4 variant identified in patients, is present at a lower steady-state level than WT murine EXOSC4 in a mouse neuronal cell line. Lysates of mouse N2a cells transfected with empty vector or vector expressing murine Myc-EXOSC4 or Myc-EXOSC4-L187P were analyzed by immunoblotting with anti-Myc antibody to detect Myc-EXOSC4 proteins. The stain-free signal serves as a loading control and neomycin phosphotransferase II (NPTII) serves as a transfection control. *B*, quantitation of the relative level of EXOSC4-L187P protein compared to EXOSC4 detected in the lysates of N2a cells expressing Myc-tagged EXOSC4 or EXOSC4-L187P from four immunoblot experiments – one shown in (*A*). The graph shows the relative level of EXOSC4-Myc protein compared to WT EXOSC4 (WT) from four independent experiments (n = 4). Error bars represent SEM. Statistical significance is calculated by a Student’s *t* test (∗∗*p*-value ≤ 0.01). *C*, Myc-EXOSC4 or Myc-EXOSC4-L187P was immunoprecipitated from N2a cells and interactions with the RNA exosome subunits EXOSC8, EXOSC9, and the RNA exosome–associated EXOSC10 were analyzed by immunoblotting. Both the input and bound samples are shown for Myc-EXOSC4 and Myc-EXOSC4-L187P. NPTII represents the neomycin phosphotransferase II, which is encoded on the Myc-EXOSC4/EXOSC4-L187P plasmids, indicating similar levels of transfection for EXOSC4 and EXOSC4-L187P. Hsp90 serves as a loading control.

Our results modeling the novel, disease-associated EXOSC4-L187P variant in budding yeast show that the L187P amino acid substitution in Rrp41 decreases the steady-state level of the protein as shown in Figure 3, *B* and *C*. To assess whether this amino acid change alters the steady-state level of the mammalian EXOSC4 protein, we expressed Myc-tagged, WT mouse EXOSC4 or EXOSC4-L187P in cultured Neuro2A (N2a) cells and examined the levels of protein by immunoblotting (Fig. 7*A*). As shown in Figure 7*A* and quantitated in Figure 7*B* and similar to what is observed for yeast Rrp41-L187P (Fig. 3, *B* and *C*), the level of the EXOSC4-L187P protein is decreased to ∼40% of the control EXOSC4 protein level, suggesting that the L187P amino acid substitution could affect the stability of the EXOSC4 protein.

To extend this analysis, we assessed the interaction of EXOSC4-L187P with other components of the RNA exosome. We immunoprecipitated Myc-tagged EXOSC4 or EXOSC4-L187P from transfected N2a cells and immunoblotted to detect other endogenous subunits of the RNA exosome complex (Fig. 7*C*). As shown in the bound lanes, similar levels of Myc-EXOSC4 and Myc-EXOSC4-L187P were immunoprecipitated under the conditions used. With WT EXOSC4, we detect copurification of two of the other core, barrel subunits, EXOSC8 and EXOSC9 (see Fig. 2*A*), and an RNA exosome-associated exonuclease, EXOSC10 (39) (Fig. 7*C*). In contrast, with EXOSC4-L187P, we observe a drastic decrease in the copurification of EXOSC8, EXOSC9, and EXOSC10, compared to control EXOSC4 (Fig. 7*C*). The input samples show that the levels of both Myc-tagged EXOSC4/EXOSC4-L187P and other proteins analyzed are similar under the conditions employed for this experiment. These results indicate that EXOSC4-L187P does not efficiently associate with other RNA exosome subunits and suggest that EXOSC4-L187P does not assemble into a stable RNA exosome to the same extent as WT EXOSC4.

## Discussion

Here, we report for the first time a pathogenic variant in *EXOSC4*, which encodes a structural subunit of the RNA exosome. This work adds the *EXOSC4* gene to a growing list of genes that are linked clinically to a class of syndromes termed RNA exosomopathies. Consistent with many of the exosomopathies reported thus far (10, 15), the pathogenic variant identified in *EXOSC4* (p.L187P) causes a single amino acid change in an evolutionary conserved sequence within the EXOSC4 subunit. Modeling of this pathogenic amino acid substitution in the budding yeast protein, Rrp41, corresponding to EXOSC4, reveals that cells expressing the Rrp41-L187P variant as the sole copy of the essential Rrp41 protein show severe growth defects as well as accumulation of a number of RNA exosome target transcripts, including 7S pre-rRNA. The *rrp41-L187P* cells also show a decrease in total polysomes along with the incorporation of 7S pre-rRNA into polysomes, suggesting a potential mechanism contributing to pathology in cells with defective RNA exosome function.

Consistent with a global requirement for the RNA exosome to support key cellular functions, such as maturation of rRNA to produce ribosomes, the phenotype we describe consists of defects in a number of different organs as well as failure to thrive. Many RNA exosomopathies are associated with both cerebellar defects and motor neuron pathology (10, 13, 20, 40). The individuals described here show upper motor neuron disease features. Why pathogenic variants that impair RNA exosome function cause tissue- or cell-specific consequences is not yet clear as the requirement for RNA exosome function in different cell types and developmental programs is only starting to be defined (41, 42, 43). Initial studies suggest that the function of the RNA exosome is critical to ensure proper differentiation of a number of different cell types (41, 43, 44, 45).

As in many clinical reports associated with pathogenic variants in RNA exosome genes (10), a single amino acid change within a core structural subunit of the RNA exosome causes pathology in the case reported here. Such single amino acid changes can alter protein function through multiple mechanisms. While the L187P change in EXOSC4/Rrp41 is not predicted to alter the overall structure of either the individual protein or the RNA exosome complex based on modeling in AlphaFold, experimental evidence from both budding yeast (Fig. 3, *B* and *C*) and cultured mammalian cells (Fig. 7, *A* and *B*) shows that the steady-state level of Rrp41-L187P/EXOSC4-L187P is decreased relative to control Rrp41/EXOSC4 protein. Studies that analyzed steady-state levels of RNA exosome subunits in RNA exosomopathy patient-derived cells show decreased levels of not only the RNA exosome subunit affected by the disease-causing variant but also other subunits (20, 22). However, the simplest model that all pathology results from an overall drop in the activity of the RNA exosome due to a decrease in the cellular levels of the complex or individual subunits seems insufficient to explain the diverse clinical presentations in patients. To provide insight into whether defects in RNA exosome function can be explained solely by a decrease in the steady-state level of the defective subunit and perhaps associated subunits, we performed an experiment to substantially increase the levels of the Rrp41-L187P protein (Fig. 4). Results of this analysis reveal that an increase in the steady-state level of the Rrp41-L187P variant does improve both cell growth and RNA exosome function, but not to the same extent as control Rrp41. These findings support a model where pathological consequences result from some combination of a decrease in levels of the defective RNA exosome subunit and specific consequences due to the pathologic amino acid change.

Building on the idea that the specific pathogenic amino acid change impacts RNA exosome function, we assessed the interaction between EXOSC4-L187P and other RNA exosome subunits, which revealed that these interactions are severely decreased (Fig. 7*C*). This finding suggests that EXOSC4-L187P does not assemble/associate with the RNA exosome complex to the same extent as WT EXOSC4. Given that Leu187 lies in close proximity to the interface with EXOSC9 (Fig. 2*C*), the change to proline at this position could disrupt this interaction, which may be important for the overall integrity of the complex. In fact, to produce recombinant proteins for structural studies, EXOSC4 and EXOSC9 are typically co-expressed (46). This proline substitution does occur within an α-helix (Fig. 2, *C* and *D*), suggesting local structural changes, consistent with a reproducible shift in migration observed for Rrp41-L187P and EXOSC4-L187P compared to the corresponding WT proteins (Figs. 3*B* and 7*A*). Taken together, these findings strongly suggest that functional levels of the RNA exosome complex are decreased at least in this mammalian cell model. A caveat of this conclusion is that in this experimental system, mammalian cells express both the WT EXOSC4 protein and the exogenous Myc-tagged EXOSC4 protein. In the future, experiments employing mammalian cells engineered to allow the analysis of EXOSC4-L187P as the sole copy of EXOSC4 as in the budding yeast model would be valuable.

This study extends the analysis of RNA exosome target transcripts to explore the consequences for translating ribosomes. Maturation of the 3′ end of the 5.8S rRNA from the 7S pre-rRNAs involves a series of cleavage steps by different nucleases (35). The trimming of 7S to 6S pre-rRNA happens in the nucleus, whereas the final processing of 6S pre-rRNA to 5.8S rRNA is cytoplasmic (37). The 3′-end trimming of the 7S pre-rRNA to the mature 5.8S (36) is one of the best defined functions of the RNA exosome complex and defects in this process can alter ribosome biogenesis and translation (47). Indeed, a recent study demonstrated that several different budding yeast models of exosomopathies impair translation, albeit, there appear to be differences in how translation is affected in different yeast exosomopathy models (48). The analysis of the core Rrp41 subunit here is consistent with previous findings (47, 48) that suggest pathogenic variants that affect the core subunits *versus* the cap subunits of the RNA exosome cause distinct translational defects. In particular, defects in core subunits, such as Rrp41, cause an overall reduction of ribosomes, while defects in cap subunits lead to the formation of halfmers, indicating a potential problem in 60S maturation or subunit joining (48). Further studies will be required to determine whether these changes in polysomes in budding yeast exosomopathy models extend to patient cells or mammalian disease models, as well as how these molecular changes alter the proteome. Such studies would be key to understanding how fundamental changes in RNA exosome function and the downstream consequences of these changes are linked to clinical consequences associated with exosomopathies.

In summary, the work presented here expands the group of disorders termed RNA exosomopathies by reporting a neurodevelopmental disorder in two sisters homozygous for a pathogenic missense variant in the *EXOSC4* gene. Studies exploring the consequences of this pathogenic amino acid substitution (L187P) in a budding yeast model reveal defects in the function of the RNA exosome, including dysregulation of translating ribosomes. A major challenge remains defining how an essential complex that contributes to critical biology in all cell types causes distinct pathological consequences.

## Experimental procedures

### Human subjects

This study was approved by the Medical Research Ethical Committee of the Sultan Qaboos University (SQU MREC#1362). Parents provided written informed consent to participate and to publish their family pedigrees and clinical data. Clinical investigations were conducted according to the principles expressed in the Declaration of Helsinki. Genetic analyses were performed in accordance with bioethics rules of national laws.

### Exome sequencing

We performed whole-exome sequence analysis on DNA isolated from blood from the two affected siblings and the father as a trio-exome analysis. DNA was barcoded and enriched for the coding exons of targeted genes using hybrid capture technology (Agilent SureSelect Human All-exons-V6). Prepared DNA libraries were then sequenced using next-generation sequencing technology [NovaSeq6000, 150 bp paired-end, at 200X coverage]. The reads were mapped against UCSC GRCh37/hg19 by Burrows-Wheeler Aligner (BWA 0.7.12). Genome Analysis Tool Kit (GATK 3.4) was used for variant calling. Variant filtration was applied to keep novel or rare variants (≤1%). Publicly available variant databases (1000 Genomes, Exome Variant Server, and GnomAD) and an in-house database of 1564 exomes were used to filter out common or benign variants specific to the Omani population. Only coding or splicing variants were considered. The phenotype and mode of inheritance (autosomal recessive) were considered. Variants of high impact or highly damaging missense, a Combined Annotation Dependent Depletion score ≥20, and shared between the affected individuals were prioritized. Sanger sequencing was used to confirm segregation. The *EXOSC4* forward and reverse primer sequences (AM1 & AM2) used for amplification of the target on *EXOSC4* are listed in Table 1.

**Table 1:** DNA oligonucleotide primers and probes

Description	Sequence (5′-3′)	Name
*rrp41-L187P* SDM Fwd	GAAGAAAATGCTATGAGTACAGTGACA**CCA**GGTGTGGTAGGGAAGTCAGAAAAAC	AC9500
*rrp41-L187P* SDM Rev	GTTTTTCTGACTTCCCTACCACACC**TGG**TGTCACTGTACTCATAGCATTTTCTTC	AC9501
*Exosc4-L187P* SDM Fwd	GCTGGAGGGCCCCAGCTCGCC**CCG**GCCCTGCTGCCCGCCTCCGGCC	AC9505
*Exosc4-L187P* SDM Rev	GGCCGGAGGCGGGCAGCAGGGC**CGG**GGCGAGCTGGGGCCCTCCAGC	AC9506
*RRP41_565-gRNA* Fwd	ATATGCATGC**GAGTACAGTGACACTAGGTG**GTTTTAGAGCTAGAAATAGC	AC9891
*SUP4t-crRNA* Rev	ATATGGTACCAGACATAAAAAACAAAAAAAGCACCACC	AC6809
*rrp41-L187P*-HDR Fwd	TACCAATTCATTAGAAGAAAATGCTATGAGTACAGTGACA**CCA**GGTGT**A**GTAGG**T**AAGTCAGAAAAACTTTCTCTTTTATTGGTGGAA	AC9893
*rrp41-L187P*-HDR Rev	TTCCACCAATAAAAGAGAAAGTTTTTCTGACTT**A**CCTAC**T**ACACC**TGG**TGTCACTGTACTCATAGCATTTTCTTCTAATGAATTGGTA	AC9894
*EXOSC4* Exome Seq Fwd	GGAAGGGTGTGATGGGGTT	AM1
*EXOSC4* Exome Seq Rev	GTGGGCAGAGGAGGGTTTTA	AM2
*U4* pre-snRNA Fwd	AAAGAATGAATATCGGTAATG	AC5722
*U4* pre-snRNA Rev	ATCCTTATGCACGGGAAATACG	AC5723
*TLC1* pre-RNA Fwd	GTATTGTAGAAATCGCGCGTAC	AC7593
*TLC1* pre-RNA Rev	CCGCCTATCCTCGTCATGAAC	AC7594
*U14* snoRNA (snR128) Fwd	GATCACGGTGATGAAAGACTGG	AC5397
*U14* snoRNA (snR128) Rev	CTACAGTATACGATCACTCAGACATCCTA	AC5398
*ALG9* mRNA Fwd	CACGGATAGTGGCTTTGGTGAACAATTAC	AC5067
*ALG9* mRNA Rev	TATGATTATCTGGCAGCAGGAAAGAACTTGGG	AC5068
P1 Probe (between A2 & A3 sites)	TGTTACCTCTGGGCCC	HG245
P2 Probe (between C2 & E sites)	GGCCAGCAATTTCAAGTTA	HG242
P3 Probe (5.8S rRNA)	CTGCGTTCTTGATCGATGCG	HG247
P4 Probe (between D & A2 sites)	GCTCTCATGCTCTTG CC	HG240
P5 Probe (18S rRNA)	CATGGCTTAATCTTTGAGAC	HG243
P6 Probe (25S rRNA)	GCCCGTTCCCTTGGCTGTG	HG244
scR1 Probe	ATCCCGGCCGCCTCCATCAC	HG315
5S Probe	CTACTCGGTCAGGCTC	HG246

The bolded nucleotides indicate codon mutations or PAM site mutations. The bolded sequence in *RRP41_565-gRNA Fwd* oligonucleotide is the gRNA sequence. The underlined sequences are restriction sites.

### Chemicals and media

All chemicals were obtained from Sigma-Aldrich, United States Biological, or Thermo Fisher Scientific unless otherwise noted. All media were prepared by standard procedures (49).

### Protein structure analysis

We used cryo-EM structures of the human RNA exosome complex [PDB: 6D6R; (27)] and the budding yeast RNA exosome complex [PDB: 6FSZ; (30)]. The PyMOL Molecular Graphics System, Version 2.0 Schrödinger, LLC was used for viewing structures and figure making. AlphaFold was used for structure predictions of the variants (28, 29).

### *S. cerevisiae* strains and plasmids

All DNA manipulations were performed according to standard procedures (50). *Saccharomyces cerevisiae* strains and plasmids used in this study are listed in Table 2. The haploid *rrp41Δ* yeast strain (yAV2493) was generated by the transformation of a heterozygous diploid *RRP41/rrp41Δ* yeast strain (BY4743 strain background; Open Biosystems) with a WT *RRP41 URA3 CEN6* maintenance plasmid and sporulation of the diploid to yield *rrp41Δ* haploid progeny containing the *RRP41* maintenance plasmid. The WT *RRP41 LEU2 CEN6* plasmid (pAC4179) and *RRP41 LEU2* 2μ plasmid (pAC2971) were generated by PCR amplification of the *RRP41* gene containing its endogenous promoter, 5′UTR, and 3′UTR from yeast genomic DNA using yeast gene-specific primers (Integrated DNA Technologies) and cloning into pRS315 (51) and pRS425 (52), respectively. The *RRP41-Myc LEU2 CEN6* plasmid (pAC4242) was generated by PCR amplification of the *RRP41* promoter/coding sequence and *2xMyc*-*RRP41* 3′UTR products using the *RRP41* plasmid (pAC4179) and yeast gene-specific primers, one of which encoded the 2xMyc tag, and cloning into pRS315 (51). The *rrp41-L187P LEU2 CEN6* plasmid (pAC4180), *rrp41-L187P-Myc LEU2 CEN6* plasmid (pAC4243), and *rrp41-L187P LEU2 2μ* plasmid (pAC4397) were generated by site-directed mutagenesis of the *RRP41 LEU2 CEN6* plasmid (pAC4179), *RRP41-Myc LEU2 CEN6* plasmid (pAC4242), and *RRP41 LEU2 2μ* (pAC2971) plasmid, respectively, using oligonucleotides encoding the L187P amino acid change (AC9500 & AC9501; Integrated DNA Technologies) and QuickChange II SDM Kit (Agilent). The *RRP41-Myc LEU2 2μ* plasmid (pAC4398) and *rrp41-L187P-Myc LEU2 2μ* plasmid (pAC4399) were generated by the excision of *RRP41-Myc* from pAC4242 and *rrp41-L187P-Myc* from pAC4243 using BamHI and ApaI and cloning into pRS425 cut with BamHI and ApaI. The pcDNA3-Myc-*Exosc4* (pAC3517) plasmid was generated by PCR amplification of the mouse *Exosc4* coding sequence from Neuro-2a cDNA using gene-specific primers (Integrated DNA technologies) and cloning into pcDNA3 (Invitrogen) plasmid containing pCMV promoter and N-terminal Myc tag. The pcDNA3-Myc-*Exosc4-L187P* (pAC4231) variant plasmid was generated by site-directed mutagenesis of pcDNA3-Myc-*Exosc4* (pAC3517) plasmid using oligonucleotides encoding the L187P amino acid change (AC9505 & AC9506; Integrated DNA Technologies) and QuickChange II SDM Kit (Agilent).

**Table 2:** *Saccharomyces cerevisiae strains* and plasmids

Strain/Plasmid	Description	Reference
BY4741 (ACY402)	*MATa ura3Δ0 leu2Δ0 his3Δ1 met15Δ0*	(60)
*rrp41Δ* (yAV2493)	*MAT? ura3Δ0 leu2Δ0 his3Δ1 rrp41Δ::NEO* [*RRP41, URA3*]	This study
*rrp41-L187P* (ACY3125)	*MATa ura3Δ0 leu2Δ0 his3Δ1 met15Δ0 rrp41-L187P*	This study
*rrp41-L187P* (ACY3126)	*MATa ura3Δ0 leu2Δ0 his3Δ1 met15Δ0 rrp41-L187P*	This study
pRS315	*CEN6, LEU2, amp*^*R*^	(51)
pRS425	*2μ, LEU2, amp*^*R*^	(52)
pAC2971	*RRP41* in pRS425, *LEU2, 2μ*	This study
pAC4179	*RRP41-Native 3′UTR* in pRS315*, CEN6, LEU2, amp*^*R*^	This study
pAC4180	*rrp41-L187P-Native 3′UTR* in pRS315*, CEN6, LEU2, amp*^*R*^	This study
pAC4242	*RRP41-2xMyc-Native 3′UTR* in pRS315*, CEN6, LEU2, amp*^*R*^	This study
pAC4243	*rrp41-L187P-2xMyc-Native 3′UTR* in pRS315*, CEN6, LEU2, amp*^*R*^	This study
pAC4397	*rrp41-L187P-Native 3′UTR* in pRS425*, 2μ, LEU2, amp*^*R*^	This study
pAC4398	*RRP41-2xMyc-Native 3′UTR* in pRS425*, 2μ, LEU2, amp*^*R*^	This study
pAC4399	*rrp41-L187P-2xMyc-Native 3′UTR* in pRS425*, 2μ, LEU2, amp*^*R*^	This study
pcDNA3	*pCMV, NeoR, amp*^*R*^	Invitrogen
pAC3517	*Myc-Exosc4* in pcDNA3, *NeoR*, *amp*^*R*^	This study
pAC4231	*Myc-Exosc4-L187P* in pcDNA3, *NeoR*, *amp*^*R*^	This study
pAC3846	*TEF1p-Cas9-CYC1t-SNR52p* in pRS316, *CEN6, URA3, amp*^*R*^	(48)
pAC4339	*TEF1p-Cas9-CYC1t-SNR52p-RRP41_565.gRNA-SUP4t* in pRS316, *CEN6, URA3, amp*^*R*^	This study

The pRS316-*TEF1p-Cas9-CYC1t-SNR52p* (pAC3846) pCas9 plasmid (*URA3*, *CEN6*), which derives from p414-*TEF1p-Cas9-CYC1t* plasmid [Addgene #43802; (53)] and p426-*SNR52p-gRNA.CAN1.Y-SUP4t* plasmid [Addgene #43803; (53)] has been described previously (48). The pRS316-*TEF1p-Cas9-CYC1t-SNR52p-RRP41_565.gRNA-SUP4t* (pAC4339) pCas9 plasmid containing the gRNA for targeting *RRP41* was constructed by the PCR amplification of *RRP41_565.gRNA* with oligonucleotides AC9891 and AC6809 using p426-*SNR52p-gRNA.CAN1.Y-SUP4t* plasmid template (Addgene #43803) and cloning of SphI/KpnI-digested gRNA product into pAC3846 digested with SphI/KpnI. All plasmids were sequenced to ensure the presence of desired mutations and absence of any other mutations.

### Generation of integrated *rrp41-L187P* mutant strains using *CRISPR-Cas9* genome editing

Two *rrp41-L187P* (ACY3125; ACY3126) mutant strains were generated using CRISPR/Cas9 editing with a single pCas9-gRNA expression plasmid and double-stranded homology-directed repair (HDR) oligonucleotides in a WT BY4741 strain essentially as described before (53). The single pCas9-gRNA plasmid on a pRS316 (*URA3, CEN6*) backbone is derived from p414-*TEF1p-Cas9-CYC1t* plasmid (Addgene #43802) and p426-*SNR52p-gRNA.CAN1.Y-SUP4t* plasmid (Addgene #43803) (53). Constitutive expression of Cas9 is driven by the *TEF1* promoter and constitutive expression of the gRNA is driven by the *SNR52* promoter. Specifically, 500 ng of pAC3846 (pCas9 without gRNA), pAC4339 (pCas9 + *RRP41* gRNA) ± 1 nmol of double-stranded *rrp41-L187P* HDR oligonucleotide (AC9893/9894), and 50 μg salmon sperm DNA was transformed into WT BY4741 cells by standard lithium acetate transformation protocol (49). HDR oligonucleotides are listed in Table 1. Cells were plated on Ura^-^ media plates and incubated at 30 °C for 2 days. Large colonies on plates with cells transformed pCas9-gRNA and HDR oligonucleotides were restreaked to new Ura^-^ media plates and screened for the presence of *rrp41-L187P* mutations *via* Sanger sequencing of PCR-amplified *RRP41*.

### *S. cerevisiae* cell growth assays

The *rrp41Δ* yeast strain (yAV2493) was transformed with WT *RRP41 LEU2 CEN6* low copy plasmid (pAC4179), *rrp41-L187P* variant *LEU2 CEN6* low copy plasmid (pAC4180), WT *RRP41 LEU2 2μ* high copy plasmid (pAC2971), or *rrp41-L187P* variant *LEU2 2μ* high copy plasmid (pAC4397) and selected on Leu- plates. The Leu+ transformants were streaked and grown on a 5-flouroorotic acid plate to select for cells that had lost the *RRP41 URA3* maintenance plasmid (54) and thus only contained the low copy *RRP41 LEU2 CEN6* plasmid, low copy *rrp41-L187P LEU2 CEN6* plasmid, high copy *RRP41 LEU2 2μ* plasmid, or high copy *rrp41-L187P LEU2 2μ* plasmid. The growth of the *rrp41Δ* cells containing only *RRP41 LEU2 CEN6*, *rrp41-L187P LEU2 CEN6, RRP41 LEU2 2μ*, or *rrp41-L187P LEU2 2μ* plasmid was assayed on solid media by growth of cells in 2 ml Leu- media overnight at 30 °C, serial dilution of the cells (in 10-fold dilutions), spotting of cells on Leu- media plates, and incubation of the plates at 25 °C, 30 °C, and 37 °C for 2 to 3 days. The *rrp41-L187P* CRISPR mutant strains (ACY3125 & ACY3126) transformed with vector (pRS425) or *RRP41* (pAC2971) 2μ *LEU2* plasmid and WT (BY4741) cells transformed with vector (pRS425) were grown in 2 ml Leu- media overnight at 30 °C and similarly serially diluted, spotted onto Leu- plates, and incubated at 25 °C, 30 °C and 37 °C for 2 to 3 days as indicated. For growth in liquid culture, cells were grown in 2 ml Leu- minimal media overnight at 30 °C to saturation, diluted to an A_600_ = 0.05 in Leu minimal media in a 24-well plate, and growth at 30 °C was monitored and recorded at A_600_ in a BioTek Synergy Mx microplate reader with Gen5 v2.04 software over 24 h. Nine technical replicates of each strain were measured, and the average of these replicates was calculated and graphed.

### Immunoblotting

For the analysis of C-terminally Myc-tagged WT Rrp41 and Rrp41-L187P variant protein expression levels from low copy (*CEN*) plasmids (in Fig. 3, *B* and *C*), triplicates of *rrp41Δ* (yAV2493) cells containing only WT *RRP41-Myc* (pAC4242) or *rrp41-L187P-Myc* (pAC4243) *LEU2 CEN6* plasmid were grown in 2 ml Leu- media overnight at 30 °C to saturation and 10 ml cultures with an A_600_ = 0.4 were prepared and grown at 30 °C or 37 °C for 5 h. For the analysis of C-terminally Myc-tagged WT Rrp41 and rrp41-L187P variant protein expression levels from both low copy (*CEN*) and high copy (*2μ*) plasmids (in Fig. 4, *A* and *B*), duplicates of *rrp41Δ* (yAV2493) cells containing only WT *RRP41-Myc* (pAC4242) or *rrp41-L187P-Myc* (pAC4243) *LEU2 CEN6* plasmid or WT *RRP41-Myc* (pAC4398) or *rrp41-L187P-Myc* (pAC4399) *LEU2 2μ* plasmid were grown in 5 ml Leu- media overnight at 30 °C to saturation and 50 ml cultures with an A_600_ = 0.1 were prepared and grown at 30 °C or 37 °C to an A_600_ = 0.8. Cell pellets were collected by centrifugation at 1962*g* for 3 min, transferred in 1 ml water to 2 ml screw-cap tubes and centrifuged at 16,000*g* for 1 min, aspirated, frozen in liquid nitrogen, and stored at −80 °C. For the analysis of EXOSC4 expression levels, mouse N2a cells (55) were transiently transfected with pcDNA3 vector (Invitrogen) containing mouse WT *Myc-Exosc4* (pAC3517) or *Myc-Exosc4-L187P* variant (pAC4231) plasmid or empty vector (pcDNA3; Invitrogen) using Lipofectamine 2000 (Invitrogen), and cells were collected by centrifugation at 2000*g* for 2 min 24 h after transfection.

Budding yeast cell lysates were prepared by the resuspension of cells in 0.5 to 1 ml RIPA-2 buffer [50 mM Tris–HCl, pH 8; 150 mM NaCl; 0.5% sodium deoxycholate; 1% NP40; 0.1% SDS] supplemented with protease inhibitors [1 mM PMSF; Pierce Protease Inhibitors (Thermo Fisher Scientific)], addition of 300 μl glass beads (425–600 μm diameter (Sigma)), disruption in a Mini Bead Beater 16 Cell Disrupter (Biospec) for 4 × 1 min at 25 °C, with cooling on ice for 1 min in between disruptor runs, and centrifugation at 16,000*g* for 10 min at 4 °C. Mouse N2a cell lysates were prepared by lysis in RIPA-2 buffer and centrifugation at 16,000*g* for 10 min at 4 °C. Protein lysate concentration was determined by Pierce BCA Protein Assay Kit (Life Technologies). Whole cell lysate protein samples (20–50 μg) were resolved on Criterion 4 to 20% gradient denaturing gels (Bio-Rad), transferred to nitrocellulose membranes (Bio-Rad), and Myc-tagged Rrp41 and EXOSC4 proteins were detected with anti-Myc monoclonal antibody 9B11 (1:2000; Cell Signaling Technology; Cat. 2276S). As loading controls, 3-Phosphoglycerate kinase (Pgk1) protein was detected with mouse anti-Pgk1 monoclonal antibody (1:30,000; Invitrogen; Cat. 459250), and stain-free signal (Bio-Rad) on the immunoblot was included. For transfection control, Neomycin phosphotransferase II (NPTII) expressed from *NeoR* cassette on pcDNA3*-Exosc4* vectors was detected with mouse anti-NPTII monoclonal antibody (1:1000; Cell Applications, Inc.; Cat. CP10330). Primary antibodies were detected using goat secondary antibodies coupled to horseradish peroxidase (1:3000; Jackson ImmunoResearch Inc), and enhanced chemiluminescence signals were captured on a ChemiDoc MP Imaging System (Bio-Rad).

### Quantitation of immunoblotting

The protein band intensities/areas from immunoblots were quantitated using ImageJ v1.4 software (National Institute of Health, MD; http://rsb.info.nih.gov/ij/) or ImageLab software (Bio-Rad), and mean fold changes in protein were calculated in Microsoft Excel for Mac Version 16.84 (Office 365, Microsoft Corporation). To quantitate the mean level of Rrp41-L187P-Myc variant relative to WT Rrp41-Myc in *rrp41Δ* cells incubated at 30 °C or 37 °C (in Fig. 3*C*), Rrp41-Myc and Rrp41-L187P-Myc intensity was first normalized to loading control Pgk1 intensity and then normalized to WT Rrp41-Myc intensity for each biological replicate. The mean Rrp41-L187P-Myc level at 30 °C and 37 °C was quantitated from biological triplicates. To quantitate the fold changes of high copy (2μ) *versus* low copy (*CEN*) Rrp41-Myc or rrp41-L187P-Myc variant in *rrp41Δ* cells incubated at 30 °C or 37 °C (in Fig. 4*B*), the *2μ* and *CEN* Rrp41-Myc and Rrp41-L187P-Myc intensity was first normalized to loading control Pgk1 intensity and then *2μ* Rrp41-Myc or Rrp41-L187P-Myc intensity was normalized to *CEN* Rrp41-Myc or Rrp41-L187P-Myc, respectively, for each biological replicate. The fold changes of *2μ versus CEN* Rrp41-Myc or Rrp41-L187P-Myc was quantitated from biological duplicates. To calculate the mean level of Myc-EXOSC4-L187P variant relative to WT Myc-EXOSC4 in N2a cells from four independent biological replicates, Myc-EXOSC4-L187P and Myc-EXOSC4 intensity were first normalized to stain-free signal and NPTII transfection control and then normalized to WT Myc-EXOSC4 for each immunoblot. The mean level of Rrp41-L187P-Myc relative to Rrp41-Myc, fold change of *2μ versus CEN* Rrp41-Myc or Rrp41-L187P-Myc, mean level of Myc-EXOSC4-L187P relative to Myc-EXOSC4, and SEM were calculated and graphically represented. The statistical significance of the difference in protein level of the Rrp41-L187P-Myc variant relative to Rrp41-Myc at 30 °C or 37 °C and Myc-EXOSC4-L187P relative to Myc-EXOSC4 was calculated using the Student’s *t* test on GraphPad software (Prism). The statistical significance of the fold changes of high copy (*2μ*) *versus* low copy (*CEN*) Rrp41-Myc or Rrp41-L187P-Myc variant at 30 °C or 37 °C was also calculated with the Student’s *t* test.

### Total RNA isolation from *S. cerevisiae*

To prepare *S. cerevisiae* total RNA, cells were grown in 10 ml cultures to A_600_ = 0.5 to 0.8 at 30 °C or 37 °C. Cell pellets in 2 ml screw-cap tubes were resuspended in 1 ml TRIzol (Invitrogen), 300 μl glass beads were added, and samples were disrupted in a Mini Bead Beater 16 Cell Disrupter (Biospec) for 2 min at 25 °C. For each sample, 100 μl of 1-bromo-3-chloropropane was added, the sample was vortexed for 15 s, and incubated at 25 °C for 2 min. Each sample was centrifuged at 21,130*g* for 10 min at 4 °C, and upper layer was transferred to a fresh microfuge tube. RNA was precipitated with 500 μl isopropanol and sample was vortexed for 10 s to mix. Total RNA was pelleted by centrifugation at 21,130*g* for 10 min at 4 °C. RNA pellet was washed with 1 ml of 75% ethanol, centrifuged at 21,130*g* for 5 min at 4 °C, and air dried for 15 min. Total RNA was resuspended in 50 μl diethylpyrocarbonate ((Sigma))-treated water and stored at −80 °C.

### Quantitative RT-PCR

For the analysis of *U4* pre-snRNA, *TLC1* pre-ncRNA, and *U14* snoRNA levels in WT *RRP41* and *rrp41-L187P* mutant cells, *rrp41Δ* (yAV2493) cells solely containing WT *RRP41 LEU2 CEN6* low copy plasmid (pAC4179), *rrp41-L187P* variant *LEU2 CEN6* low copy plasmid (pAC4180), WT *RRP41 LEU2 2μ* high copy plasmid (pAC2971), or *rrp41-L187P LEU2 2μ* high copy plasmid (pAC4397) were grown in biological triplicate in 2 ml Leu- media overnight at 30 °C, 10 ml cultures with an A_600_ = 0.4 were prepared, and grown at 37 °C or 30 °C for 5 h. Cells were collected by centrifugation at 1962*g* for 3 min, transferred in 1 ml water to 2 ml screw-cap tubes, and centrifuged at 16,000*g* for 1 min, aspirated, and stored at −80 ºC. Following total RNA isolation from each cell pellet, 1 μg RNA was reverse transcribed to first strand cDNA using the M-MLV Reverse Transcriptase (Invitrogen) and 0.3 μg random hexamers according to manufacturer’s protocol. Quantitative PCR was performed on technical triplicates of cDNA (10 ng) from independent biological triplicates using gene-specific primers to detect *U4* pre-snRNA, *TLC1* pre-ncRNA, and *U14* snoRNA (0.5 μM; Table 1) and QuantiTect SYBR Green PCR master mix (Qiagen) on a StepOnePlus Real-Time PCR machine (Applied Biosystems; T_anneal_ = 55°C; 44 cycles). Primers to detect *ALG9* mRNA were used as a normalization control (Table 1). The mean RNA levels were calculated by the ΔΔCt method (56), normalized to mean RNA levels in *RRP41* cells, and converted and graphed with GraphPad software (Prism) as RNA fold change relative to *RRP41* with error bars that represent the SEM.

### Northern blot analysis of rRNAs

For the analysis of ribosomal RNAs, triplicate colonies of WT cells (BY4741) and *rrp41-L187P* CRISPR mutant cells (ACY3125) were inoculated into 5 ml yeast extract, peptone, dextrose (YEPD) media and grown at 30 °C overnight to saturation. The saturated, overnight cultures were rediluted in 50 ml YEPD media to an A_600_ = 0.1 for day cultures and grown to log phase at 30 °C to an A_600_ = 0.4 to 0.6. The log phase, day cultures were rediluted in 50 ml YEPD media for final cultures to an A_600_ = 0.001 to 0.002 and grown overnight to log phase at 30 °C to an A_600_ = 0.6. The final cultures (40 ml) were collected by centrifugation at 1962*g* for 3 min, transferred in 1 ml water to 2 ml screw-cap tubes, centrifuged at 16,000*g* for 1 min, aspirated, and cell pellets were frozen in liquid nitrogen and stored at −80 °C. Total RNA was extracted using the hot phenol method (57). Northern blotting was carried out essentially as previously described (58) using the rRNA oligonucleotide probes—P1 (between the A2 and A3 sites), P2 (between the C2 and E sites), P3 (5.8S), P4 (between the D and A2 sites), P5 (18S), and P6 (25S)—listed in Table 1. Either 5S or sc*R1* RNA was used as a loading control (Table 1).

### Polysome analysis

To analyze polysome profiles, WT (BY4741) and *rrp41-L187P* CRISPR mutant (ACY3125) cells were cultured to mid-log phase in YP-dextrose media at 37 °C and harvested with or without the addition of 0.1 mg/ml cycloheximide. Subsequently, the cells were washed and lysed in an ice-cold gradient buffer composed of 20 mM Hepes, pH 7.4 (adjusted with potassium hydroxide), 5 mM MgCl_2_, 100 mM NaCl, and 2 mM DTT, supplemented with complete protease inhibitor cocktail (Roche) and 0.1 mg/ml cycloheximide or no cycloheximide. Cryogenic grinding was used to break the cells, and the resulting cell lysate was cleared by centrifugation at 10,000*g* for 10 min. The absorbance of the cleared lysate was measured at 260 nm, and an equal amount of lysate was applied to 10 to 50% sucrose gradients in gradient buffer for all samples. For samples lacking cycloheximide, 2.5 mM puromycin was added to the cleared cell lysate. The lysate was then incubated on ice for 15 min and subsequently placed at 37 °C before being loaded onto the gradient. Gradients were centrifuged in a SW41Ti rotor at 40,000 rpm for 2 h and fractionated using a BioComp fraction collector. Northern blotting was used to analyze rRNA species in polysome fractions. Northern blots were quantified using Image Lab (Bio-Rad). The intensity of 7S pre-rRNA or 5.8S rRNA bands in polysome fractions relative to total 7S pre-rRNA or 5.8S rRNA in all gradients fractions is reported. Experiments were performed using three independent yeast colonies.

### Cell culture

Neuro2a (N2a) cells are a mouse neuroblastoma cell line (59). Cells were maintained in Dulbecco's modified Eagle's medium, supplemented with 10% fetal bovine serum and antibiotics, and grown under standard environmental conditions. The authenticity of the cell line was validated using STR profiling. In addition, the cell line was tested for *mycoplasma*, and cells were free from *mycoplasma* contamination for all experiments.

### Immunoprecipitation of mammalian RNA exosome subunits

To immunoprecipitate mouse EXOSC4, murine Neuro-2a cells (N2a) were transiently transfected with *Myc-Exosc4* (pAC3517) or *Myc-Exosc4-L187P* (pAC4231) plasmid using Lipofectamine 2000 (Invitrogen), and cells were collected 48 h after transfection. Cells were lysed in Hepes-binding buffer [20 mM Hepes-NaOH, pH 7.2; 150 mM NaCl; 0.2% Triton X-100; Pierce Protease Inhibitors (Thermo Fisher Scientific)]. Cell lysates (250–300 μg) in 0.3 ml Hepes-binding buffer were incubated with 20 μl Pierce anti-c-Myc magnetic beads (Thermo Fisher Scientific) for 3 h at 4 °C with mixing. Beads were collected by placing tubes in magnetic stand, unbound supernatant was removed, and beads were washed three times with 0.3 ml binding buffer. Input (25 μg) and Bound Myc bead (1/2 total amount) samples were analyzed by SDS-PAGE and immunoblotting with rabbit anti-Myc monoclonal antibody (1:2000; Cell Signaling Technology; Cat. 2276S) to detect Myc-tagged EXOSC4, rabbit anti-EXOSC8 (1:1000; Proteintech; Cat. 11979-1-AP) to detect endogenous EXOSC8, rabbit anti-EXOSC9 (1:1000; Bethyl Laboratories, Inc.; Cat. A303-888A) to detect endogenous EXOSC9, or rabbit anti-EXOSC10 (1:1000; Bethyl Laboratories, Inc.; Cat. A303-987A) to detect endogenous EXOSC10. Mouse anti-NPTII monoclonal antibody (1:1000; Cell Applications, Inc.; Cat. CP10330) was used to detect Neomycin phosphotransferase II as a transfection control and mouse anti-HSP90 α/β monoclonal antibody (1:2000; Santa Cruz Biotechnology; sc-13119) to detect HSP90 was used a loading control. The specificity of all antibodies was confirmed either by immunoblotting cells that do not express epitope-tagged proteins or by using siRNA-mediated depletion.

## Data availability

Strains and plasmids are available upon request. The authors affirm that all data necessary for confirming the conclusions of the article are present within the article, figures, and tables.

## Supporting information

This article contains supporting information.

## Conflicts of interest

The authors declare that they have no conflicts of interest with the contents of this article.
