# Supplementary material for: A biallelic variant of the RNA exosome gene, EXOSC4, associated with neurodevelopmental defects impairs RNA exosome function and translation
Source: J Biol Chem. 2024 Jul 14;300(8):107571. doi: 10.1016/j.jbc.2024.107571 (PMC11357806; doi:10.1016/j.jbc.2024.107571)
Supplement: Supporting information [file mmc1.pdf]

**A biallelic variant of the RNA exosome gene, *EXOSC4*, associated with neurodevelopmental defects impairs RNA exosome function and translation**

Milo B. Fasken[1], Sara W. Leung[1], Lauryn A. Cureton[2,3] Maha Al-Awadi[4], Adila Al-Kindy[5], Ambro van Hoof[6], Sohail Khoshnevis[2], Homa Ghalei[2], Almundher Al-Maawali[5,7], and Anita H. Corbett[1]

Figure S1

Figure S2

Figure S3

# Figure S1

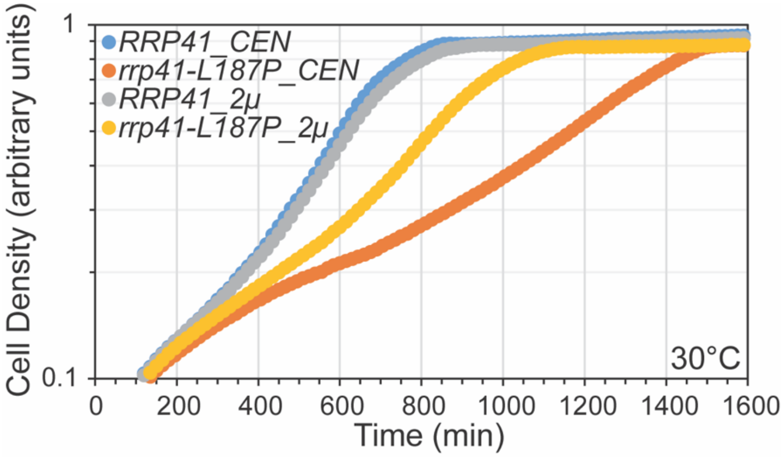


**Figure S1. Cells that express increased levels of the Rrp41-L187P protein show improved growth compared to cells with lower levels of the Rrp41-L187P protein**. Rrp41 or Rrp41-L187P protein was expressed from either a Low Copy (*CEN*) or High Copy (*2μ*) plasmid in *rrp41Δ* cells. The *rrp41*Δ cells containing *RRP41* expressed from a *CEN* (blue) or *2μ* (gray) plasmid or *rrp41-L187P* expressed from a *CEN* (orange) or *2μ* (yellow) plasmid were analyzed in a growth curve at 30°C. The graph shows a logarithmic plot of Cell Density plotted over Time in minutes.

**Figure S2**

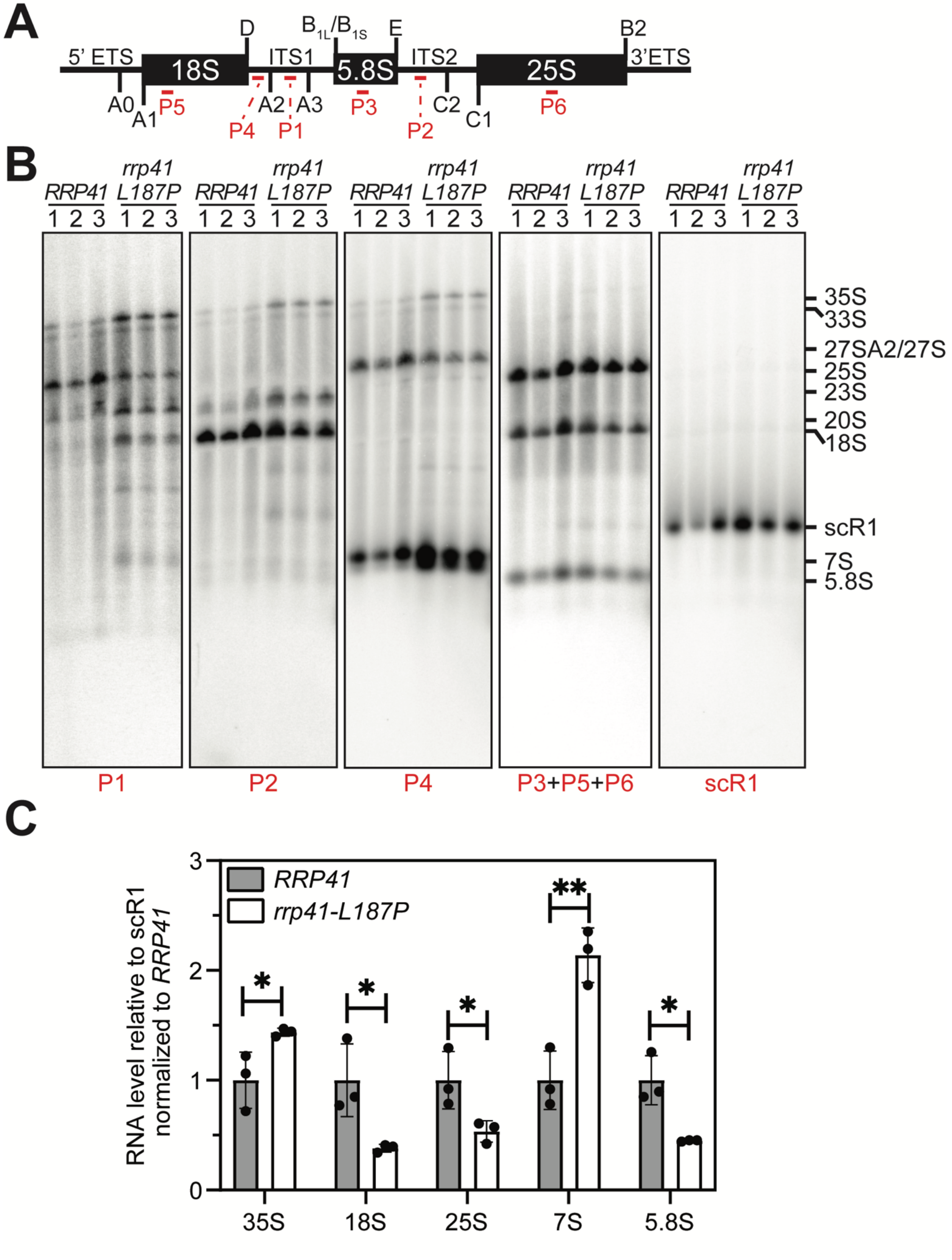
A
5' ETS
18S
D
ITS1
$B_{1L}/B_{1S}$
5.8S
E
ITS2
25S
B2
3'ETS
A0
A1
P5
P4
A2
P1
A3
P3
P2
C2
C1
P6
B
RRP41
rrp41 L187P
1 2 3 1 2 3
35S
33S
27SA2/27S
25S
23S
20S
18S
scR1
7S
5.8S
P1
P2
P4
P3+P5+P6
scR1
C
RRP41
rrp41-L187P
RNA level relative to scR1 normalized to RRP41
3
2
1
0
*
**
35S
18S
25S
7S
5.8S

**Figure S2. The *rrp41-L187P* mutant cells exhibit defects in rRNA processing**. *A*, Schematic of yeast 35S rRNA indicating the locations of rRNA cleavage sites (A-E; Black) and northern blot probes used (P1-P6; Red). The 35S rRNA transcript contains 18S, 5.8S, and 25S rRNA separated by internal transcribed spacer 1 and 2 (ITS1, ITS2) and flanked by 5' and 3' external transcribed spacer (5'ETS, 3'ETS). *B*, Equal amounts of total RNA from *rrp41*Δ cells solely expressing wild-type *RRP41* or *rrp41-L187P* grown at 30°C was used for northern blotting. To ensure the reproducibility of the data, three individual replicates are shown for each sample. *C*, To quantitate the results of northern blotting, the three samples were analyzed as described in Experimental Procedures where rRNA levels were normalized to the *scR1* transcript and the level of 35S, 18S, 25S, 7S, and 5.8S rRNA in wild-type control *RRP41* cells was set to 1.0. Statistical significance was assessed by Student's t-test.

# Figure S3

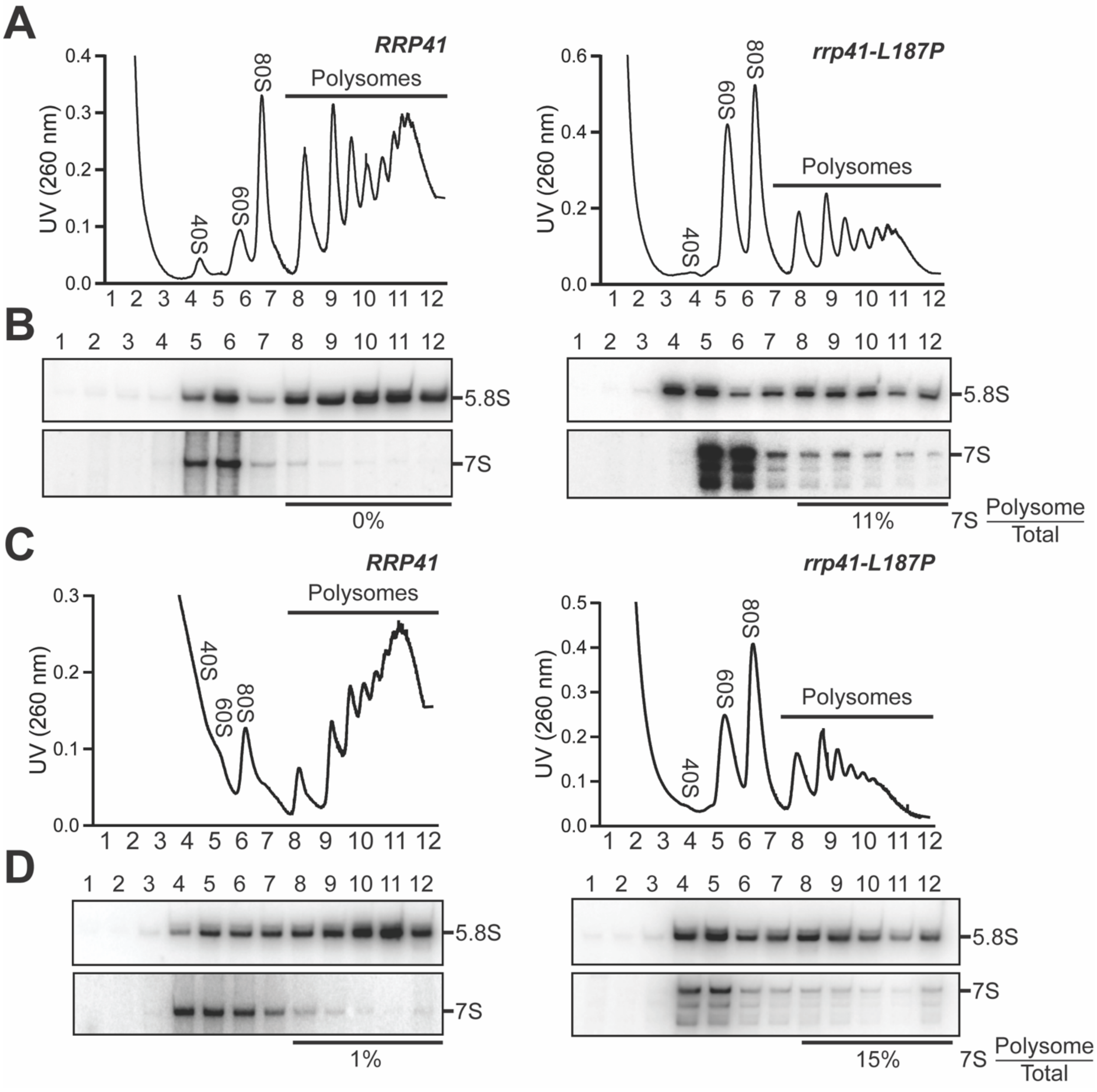


**Figure S3. The *rrp41-L187P* mutant cells show altered polysome profiles.** Two independent polysomes profiles demonstrate that polysome profile defects observed in *rrp41-L187P* CRISPR mutant cells are reproducible. *A*,*C* For two biological replicates, polysome profiling reveals that *rrp41-L187P* CRISPR mutant cells show a loss of polysomes at 37°C as compared to *RRP41* cells. Clarified cell extracts of *rrp41-L187P* CRISPR mutant cells and *RRP41* wild-type cells were

resolved on a 10-50% sucrose gradient and scanned at 260 nm. The fraction numbers are indicated and peaks corresponding to the small (40S) and large (60S) ribosome subunits, as well as the monosomes (80S) and polysomes are marked. *B,D* Northern blotting on fractions from sucrose gradients treated with cycloheximide reveal the distribution of 5.8S rRNA and 7S pre-rRNA. The fraction of 7S rRNA present in polysomes was quantitated as described in Experimental Procedures.
